# CRISPR/Cas9-Mediated Knockout of *BmGDAP2* in the Silkworm, *Bombyx mori*: Extended Lifespan and Altered Gene Expression Impacting Developmental Pathways

**DOI:** 10.3390/insects16040354

**Published:** 2025-03-27

**Authors:** Chaojun Yuan, Zichong Zhou, Qifeng Guo, Ying Yang, Yue Sun, Yong Liu, Wenyi Jia, Shuoqi Fan, Jinxin Wu, Xiaoting Hua, Ping Lin, Ping Zhao, Qingyou Xia

**Affiliations:** Integrative Science Center of Germplasm Creation in Western China (Chongqing) Science City, Biological Science Research Center, Southwest University, Chongqing 400715, China; ycj908120901@126.com (C.Y.); zzc1033408219@email.swu.edu.cn (Z.Z.); f1610639542@email.swu.edu.cn (Q.G.); spacecraft4466@email.swu.edu.cn (Y.Y.); sy981112@email.swu.edu.cn (Y.S.); liuyong20221019@126.com (Y.L.); jwy10480202@email.swu.edu.cn (W.J.); ww2023@email.swu.edu.cn (S.F.); bhwjx1993@swu.edu.cn (J.W.); huaxiaotingswu@126.com (X.H.); linpingswu@swu.edu.cn (P.L.); zhaop@swu.edu.cn (P.Z.)

**Keywords:** *GDAP2*, silkworm, development, lifespan

## Abstract

Ganglioside-induced differentiation-associated protein 2 (*GDAP2*) is a gene involved in hereditary cerebellar ataxia. At present, little is known about the function of *GDAP2* in insects. In this study, *BmGDAP2* was detected to be highly expressed in the head, epidermis, midgut, and anterior silk glands of silkworms. We generated a knockout mutant, *BmGDAP2* (*BmGDAP2*^KO^), using the CRISPR/Cas9 system. Compared with that of the wild-type, the growth cycle of *BmGDAP2*^KO^ larvae was significantly prolonged, while their body size was reduced. Furthermore, we found 149 differentially expressed genes (DEGs) between *BmGDAP2*^KO^ and the wild-type, including 106 upregulated and 43 downregulated genes. GO annotation analysis indicated that *BmGDAP2* primarily influences structural and molecular activities, as well as catalytic and binding functions. KEGG pathway analysis revealed that the differentially expressed genes were mainly enriched in pathways related to peroxidase activity, hormone synthesis, apoptosis, and longevity regulation. Further investigation focused on candidate genes related to these pathways. We found that the expression levels of *MAD2L1*, which can inhibit cell proliferation and promote apoptosis, and *Aurka*-b, which plays a crucial role in cell cycle regulation, were significantly reduced in *BmGDAP2*^KO^ silkworms. These changes may interfere with the normal functions of cell division, leading to the prolonged developmental cycle observed in *BmGDAP2*^KO^ larvae. Our findings demonstrate that knockout of *BmGDAP2* significantly prolongs the life cycle of *Bombyx mori* by affecting genes related to autophagy, apoptosis, and hormone regulation.

## Simple Summary

The life cycle of the silkworm is a complex and delicate developmental process regulated by a variety of internal and external factors, including hormones, gene expression, and environmental conditions. *GDAP2* has been proven to play a crucial role in neuronal differentiation. In this study, we used CRISPR/cas9 technology to knock out the *BmGDAP2* gene in silkworms, leading to slower development and an extended lifespan. Further transcriptome analysis showed that this is mainly because this knockout affects the expression of genes related to autophagy and apoptosis, hormone regulation, cell division, and the peroxisome pathway, thus influencing the growth and development of the silkworm.

## 1. Introduction

The complete life cycle of the silkworm is characterized by four distinctive developmental stages, including embryo (egg), larva (caterpillar), pupa, and moth (adult) phenotypic metamorphosis within each generation [1,2,3]. After oviposition, diapause-destined eggs gradually enter diapause for 10 days, with a series of dramatic changes occurring during the onset of diapause [4]. In general, silkworm larvae are tetramolters that proceed through four instars, molting between each instar [5]. The first instar lasts approximately 4 days, the second instar 3 days, followed by the third and fourth instar, each spanning 4 days, and the fifth instar of the silkworm larva is a period of high mulberry consumption and lasts approximately 6–7 days. When silkworms stop eating, they begin to spin cocoons to wrap themselves, which is the wandering stage. After 2 or 3 days, the silkworms finish the transformation from larvae to pupae in the cocoons. The newborn pupae have a soft and faint yellow cuticle. Several hours later, the cuticle becomes restrictive and rigid. After about 10 days, the silk moths break the chitinous cover and finish the transition from pupa to moth [6]. Hormones, gene expression, and environmental conditions have been reported as key factors in the developmental process [7,8,9,10]. However, due to the lack of studies on silkworm’s developmental stages, there remains a lack of understanding regarding silkworm growth and cultivation.

The ganglioside-induced differentiation-associated protein (*GDAP*) family comprises ten members, *GDAP1* to *GDAP10*, and has been shown to play a crucial role in neuronal differentiation [11,12,13]. Patients with autosomal recessive cerebellar ataxia often exhibit typical symptoms of ataxia, such as unsteady walking, limb coordination difficulties, progressive spasticity, and intellectual decline [14,15]. In a mouse model of epilepsy, overexpression of *GDAP2* significantly increased the frequency of epileptic seizures [16]. In fruit flies, interference with *GDAP2* resulted in defects in righting behavior, reduced movement, and uncoordinated walking, with even a significant impairment in flight ability. Additionally, it was found that when the *GDAP2* gene was inactivated, fruit flies became more sensitive to environmental stressors such as reactive oxygen species and nutrient deprivation, indicating its important role in cellular stress responses [17]. Notably, *BmGDAP2* was found to be related to the increased silk yield and overall robustness of domestic silkworms [18]. Because the silk-spinning behavior of silkworms is a highly complex motor behavior involving fine control of the head and brain activity, it is speculated that the upregulation of *GDAP2* may be related to the increased silk yield and overall robustness of improved silkworms. However, whether *BmGDAP2* is involved in the development of silkworms is still unclear.

Given the lack of studies and the unexplained involvement of *BmGDAP2* in silkworm development, this study sought to further investigate the function of *BmGDAP2* in *Bombyx mori*. We cloned and identified the *BmGDAP2* gene and a knockout mutant for it using the CRISPR/Cas9 system. Applying RNA-seq-based transcriptome analysis, we investigated the gene expression in *BmGDAP2*^KO^ and wild-type silkworms. The results revealed the importance of many core genes in specific pathways related to metamorphosis in insects. Moreover, our study provides a comprehensive understanding of *BmGDAP2*’s roles in physiology and biochemistry. The findings shed new light on the molecular mechanisms underlying silkworm development and highlight the potential regulatory functions of *GDAP2* in other organisms.

## 2. Materials and Methods

### 2.1. Bioinformatics Analysis

The full-length cDNA and protein sequences of *BmGDAP2* (XP_062525983.1) along with the protein sequences of *GDAP2* from eight additional species—*Drosophila melanogaster* (NP_001260790.1), *Helicoverpa zea* (XP_047022468.1), *Spodoptera litura* (XP_022825685.1), *Pararge aegeria* (XP_039749491.1), *Vanessa tameamea* (XP_026496159.1), *Danaus Plexippus* (XP_061377741.1), *Mus musculus* (NP_001397040.1), and *Homo sapiens* (NP_060156.1)—were retrieved from the National Center for Biotechnology Information (NCBI) database. The protein domain architecture was analyzed using SMART (http://smart.embl-heidelberg.de/ (accessed on 10 March 2023)). Multiple sequence alignment was performed using Clustal 2.1, and the phylogenetic tree of *GDAP2* proteins from nine species was constructed with MEGA11 software (http://www.megasoftware.net (accessed on 20 March 2023)) through the neighbor-joining method.

### 2.2. Silkworm

The non-diapausing *Bombyx mori* strain D9L in this study was maintained by the Integrative Science Center of Germplasm Creation in Western China (Chongqing) Science City. *Bombyx mori* larvae were maintained at 26 ± 1 °C with a humidity level of 75% under a 12-h light/dark cycle. They were fed ad libitum with harvested mulberry leaves.

### 2.3. RNA Extraction and RT-qPCR

Total RNA was extracted using an RNA pure Rapid RNA Kit (Magen, Foshan, China) and reverse-transcribed into cDNA with M-MLV Reverse Transcriptase (Invitrogen, Waltham, MA, USA) following the manufacturer’s protocols. qRT-PCR analysis of *BmGDAP2* expression was performed in 20 μL reactions containing SYBR Premix Ex Taq on a qTOWER2.2 thermal cycler (Analytikjena Biometra, Göttingen, Germany) under the following conditions: 95 °C for 60 s (initial denaturation) and 40 cycles of 95 °C for 20 s, 60 °C for 60 s and 72 °C for 35 s. In addition, *B. mori* sw22934 was used as an internal reference gene [19], and the relative gene expression level was analyzed quantitatively using the 2^−ΔΔCt^ method. The sequences of all the primers are listed in Table S1.

### 2.4. Plasmid Construction

The exon sequence of *BmGDAP2* was selected as the target region for knockout. sgRNA was designed using CCtop (https://www.cos.uni-heidelberg.de/en (accessed on 15 May 2023)) on the first exon, followed by synthesis of the sgRNA sequence (sequence: GCCCTTTTCGTGGCTCAAGG) [20]. The synthesized sgRNA was annealed to form double-stranded sgRNA. The piggyBac (3 × P3-EGFP-SV40-U6-TTTTTT) backbone vector conserved in our laboratory was digested with the restriction endonuclease AarI. The double-stranded sgRNA was subsequently ligated into the linearized piggyBac backbone to construct the final plasmid, designated as piggyBac-(3 × P3-EGFP-SV40-U6-*BmGDAP2*_gRNA-TTTTTT).

### 2.5. Microinjection and Screening

Knockout vector plasmids and helper plasmids [21] were mixed at a 1:1 molar ratio at 600 ng/μL and injected into silkworm eggs at a volume of 0.1 μL per silkworm egg. Finally, the injected silkworm eggs were stored at 25 ± 1 °C and under 70 ± 5% humidity. Subsequently, hatched larvae (G0) were reared on fresh mulberry leaves until they reached the adult stage and sib-mated to generate the G1 generation. The G1 eggs or moths (*BmGDAP2*-sgRNA strains) were screened for enhanced green fluorescent protein (EGFP) expression driven by the neuron-specific 3 × P3 promoter by using the blue excitation light of the fluorescence microscope (Olympus, Tokyo, Japan). Finally, the positive G1 strain was crossed with the N4 strain (pBac [IE1-EGFP-Nos-Cas9] and ubiquitous Cas9 expression under a body segment-specific promoter) [22], generating positive hybrid F1 individuals, which were selected through the screening of green fluorescence markers in the eyes and segment of the late embryos and moths using the blue excitation light of the fluorescence microscope (Olympus, Tokyo, Japan).

### 2.6. Phenotypic Observation and Statistics

Transgenic silkworms were screened using a fluorescence microscope (Olympus, Tokyo, Japan), and the genome was extracted as a template. PCR amplification was performed using *BmGDAP2* target site detection primers. The PCR fragments were subjected to agarose gel electrophoresis, purified, ligated to T-vectors, and transformed into Escherichia coli, and individual clones were sequenced through the Sanger method using M13F/R primers. The wild-type and transgenic silkworms were co-reared until the initiation of the fourth instar. Daily body weight changes were recorded from the fourth instar through the pupal stage, while the duration required for completing each developmental instar was systematically documented.

### 2.7. Analyses of RNA-Seq Data

Because phenotypic differences began to appear in the third instar larvae, RNA-seq was performed at this stage to identify the key genes affected by *BmGDAP2* knockout. Third-instar day 1 *BmGDAP2*^KO^ and wild-type larvae were collected for RNA extraction. RNA extraction, cDNA library construction, and RNA sequencing were all performed by Majorbio Co., Ltd. (Shanghai, China). The raw data were filtered with the following criteria: (1) reads with ≥10% unidentified nucleotides (N); (2) reads with >10 nt aligned to the adapter, allowing ≤10% mismatches; and (3) reads with >50% bases having Phred quality <5. The clean data were mapped to the *Bombyx mori* reference genome using Tophat with a 2 nt fault tolerance and analyzed using Cufflinks [23,24]. The relative expression of each gene was calculated using the widely used fragments per kilobase of exon per million pair-end reads mapped (FPKM) [25] using Cuffdiff. In order to identify differentially expressed genes (DEGs), Cuffdiff was further used to perform pairwise comparisons between wild-type and *BmGDAP2*^KO^ samples, with a corrected *p*-value of 0 < 0.05 and Log2|foldchange| > 1. KEGG and GO enrichment analyses of DEGs were performed with an online platform (http://www.omicshare.com/tools/ (accessed on 22 October 2024)). Based on the analytical results, 11 candidate DEGs were selected for further investigation. Detailed information for all the candidate DEGs is comprehensively listed in Table S2.

### 2.8. Statistical Analysis

All the data are presented as the means ± standard deviations (SDs). Statistical significance was analyzed using Student’s *t*-test and is denoted as follows: * *p* < 0.05, ** *p* < 0.01, *** *p* < 0.001, and **** *p* < 0.0001.

## 3. Results

### 3.1. GDAP2 Exhibits High Homology and Conserved Phylogenetic Relationships and Tissue Expression Analysis

**Figure 1:**
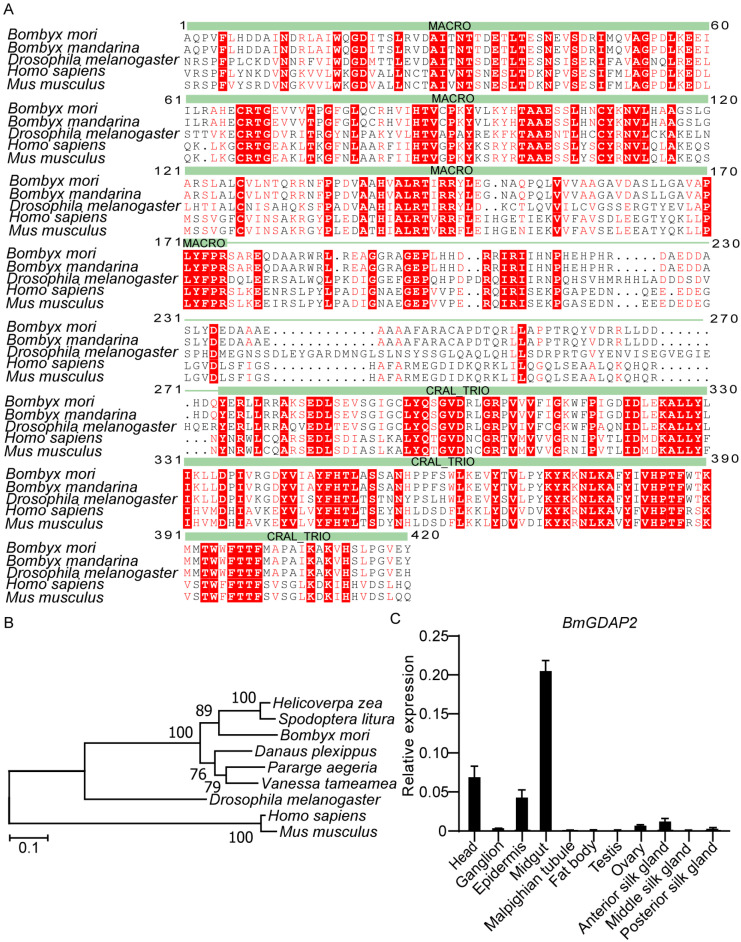
Bioinformatics analysis of *GDAP2*. (**A**) Multiple sequence alignment of *GDAP2* protein sequences across *Bombyx mori*, *Drosophila melanogaster*, *Vanessa tameamea*, *Helicoverpa zea*, and *Spodoptera litura*, highlighting conserved structural domains. (**B**) NJ phylogenetic tree of *GDAP2*s. The tree was based on multiple alignments of full-length *GDAP2* amino acid sequences from *Bombyx mori* and other species. The tree is drawn to scale. (**C**) qRT-PCR analysis of *BmGDAP2* transcripts in different tissues on day 3 fifth-instar larvae. Head, fat body, silk gland, midgut, Malpighian tubule, ovary, testis, and ganglion. Values are represented as means ± S.E.s (error bars).

To investigate the potential role of *BmGDAP2* in silkworms, we identified a sequence (XP_062525983.1) of the *GDAP2* gene that spans 1458 base pairs and consists of seven exons and six introns, encoding a putative protein comprising 485 amino acids. SMART analysis revealed conserved MACRO and CRAL_TRIO domains spanning amino acid residues 33-473 (Figure 1A). The MACRO domain, known to mediate ADP-ribosylation involved in cellular signaling and transcriptional regulation, was identified alongside the CRAL_TRIO domain. The CRAL_TRIO domain, typically found in GTPase-activating proteins (GAPs) and guanine nucleotide exchange factors (GEFs), facilitates binding to lipophilic molecules for regulatory functions.

To analyze the sequence homology, *BmGDAP2* sequences from *Bombyx mori*, *Bombyx mandarina*, *Helicoverpa zea*, *Spodoptera litura*, *Danaus Plexippus*, *Parage aegeria*, *Vanessa*, *Drosophila melanogaster*, *Mus musculus*, and *Homo sapiens* were used for phylogenetic analysis. The sequences were primarily clustered into two groups; all the sequences from mammals were clustered together, whereas *BmGDAP2* and other sequences formed the other group (Figure 1B). Although *BmGDAP2* was ubiquitously expressed in a variety of tissues of day 3 fifth-instar silkworm larvae (Figure 1C), prominent expression was noticed in the head, midgut, and epidermis; these results suggest that *BmGDAP2* may play a role in growth and development.

### 3.2. CRISPR/Cas9-Mediated Mutagenesis of BmGDAP2

**Figure 2:**
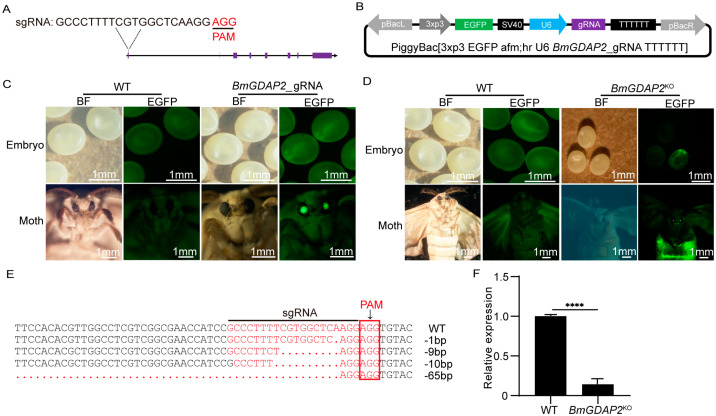
Genomic disruption of *BmGDAP2* using CRISPR/Cas9. (**A**) The sgRNA for *BmGDAP2* knockout. Each purple box represents one exon. (**B**) Schematic representation of the *BmGDAP2*-sgRNA transgenic knockout vector. (**C**) *BmGDAP2*-sgRNA expression cassettes were driven by the U6 promoter. EGFP was used as a selection marker. (**D**) Following the hybridization of *BmGDAP2*-sgRNA with N4cas9, positive F1 individuals were selected by screening for green fluorescence markers in the eyes and segments of the late embryos and moths. (**E**) Sequence alignment of sgRNA targeting genomic regions. The red arrow shows a fragment deletion. (**F**) qRT-PCR analysis of *BmGDAP2* transcripts in wild-type and *BmGDAP2*^KO^ in day 3 fifth-instar larvae. For the significance test, **** *p* < 0.0001.

To characterize the function of *BmGDAP2*, the CRISPR/Cas9 system was used to downregulate the expression of endogenous *BmGDAP2* in silkworms. A single-guide RNA (sgRNA) targeting the first exon of *BmGDAP2* was designed (Figure 2A) and ligated into a pBac-derived vector (Figure 2B). The recombinant plasmid construct was microinjected into 200 freshly laid silkworm eggs (G0 generation) within 2 h post-oviposition to generate G0. Ultimately, a total of 40 hatched larvae were successfully obtained. Surviving G0 larvae were reared to adulthood and sib-mated to generate G1 progeny. In a screening of 350 eggs from a single brood, 12 eggs exhibited enhanced green fluorescence, resulting in a positive rate of approximately 3%. The positive G1 embryos and moths expressing sgRNA (named *BmGDAP2*-gRNA) were screened for enhanced green fluorescent protein (EGFP) expression (Figure 2C). Then, the positive G1 strain was crossed with the N4 strain (Figure S1), generating positive hybrid F1 individuals, which were selected through screening for green fluorescence markers in the eyes and segment of the late embryos and moths to generate *BmGDAP2* mutants (*BmGDAP2*^KO^) (Figure 2D and Figure S2). To investigate whether *BmGDAP2* was downregulated by the CRISPR/Cas9 system, we performed PCR to amplify the sgRNA-targeted region using *BmGDAP2*-specific primers. Sequence analysis revealed the presence of deletions at the protospacer adjacent motif (PAM) site. As a result, 10 clones displayed different mutations, according to amplification and sequencing of the sgRNA target region in *BmGDAP2*^KO^ including deletions of 1, 9, 10, and 65 bp (Figure 2E). Notably, 90% of the mutant alleles exhibited frameshift mutations caused by these deletions. Quantitative real-time PCR (qRT-PCR) analysis also confirmed a significant reduction in *BmGDAP2* transcript levels in the *BmGDAP2*^KO^ larvae compared to wild-type at the fifth-instar day 3. These results demonstrate that *BmGDAP2* was successfully downregulated in the silkworms (Figure 2F).

### 3.3. Phenotypes Induced by Disruption of BmGDAP2

**Figure 3:**
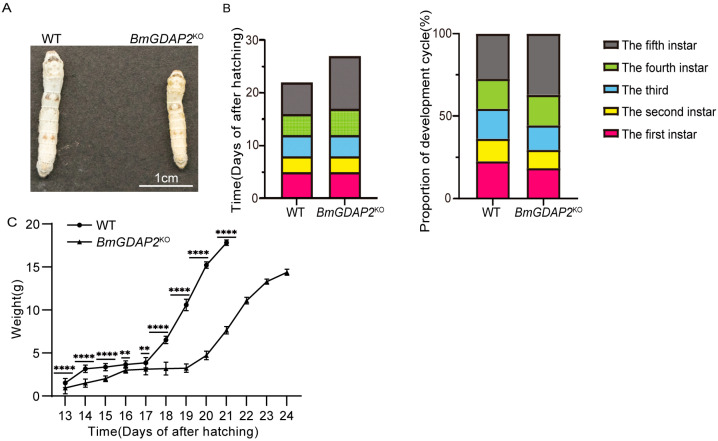
The phenotypes of *BmGDAP2* mutants. (**A**) The body size of *BmGDAP2*^KO^ larvae and WT on day 5 in the fifth instar. (**B**) The developmental time of each instar of *BmGDAP2*^KO^ larvae and WT and the proportion of each instar in the whole cycle. (**C**) Daily body weight changes of *BmGDAP2*^KO^ larvae and WT from the fourth instar to the wandering stage. Values are represented as means ± S.E.s (error bars). For the significance test, ** *p* < 0.01 and **** *p* < 0.0001.

To assess the developmental consequences of *BmGDAP2* knockout, WT and *BmGDAP2*^KO^ larvae were co-reared until cocoon spinning. At the third-instar stage, *BmGDAP2*^KO^ mutants were identified by dual green fluorescence in both eyes and body segments, while WT individuals lacked any fluorescent selection markers. There were no significant differences in developmental progression between *BmGDAP2*^KO^ and WT larvae during the first- to third-instar stages. However, *BmGDAP2*^KO^ larvae exhibited a markedly smaller body size compared to WT from the fourth instar (Figure 3A).

The developmental time from egg hatching to the wandering stage was compared between *BmGDAP2*^KO^ and wild-type larvae. It was observed that *BmGDAP2*^KO^ larvae entered the wandering stage on day 25 after hatching, while the WT entered this stage on day 22 (Figure 3B). Additionally, the proportion of the fifth-instar duration in the entire larval period was significantly increased in *BmGDAP2*^KO^ larvae compared to WT. Furthermore, the body weight of *BmGDAP2*^KO^ and WT larvae was measured from the fourth instar to the wandering stage. The results showed that the body weight of *BmGDAP2*^KO^ gradually decreased compared to that of WT, and the weight difference was most pronounced at the end of the fifth instar (Figure 3C). These findings demonstrate that the deletion of *BmGDAP2* leads to a significant elongation of the fifth-instar period in silkworm larvae, resulting in an extended developmental cycle.

### 3.4. Differentially Expressed Genes (DEGs) and Functional Enrichment Analysis from BmGDAP2^KO^-Mutant and Wild Silkworms

**Figure 4:**
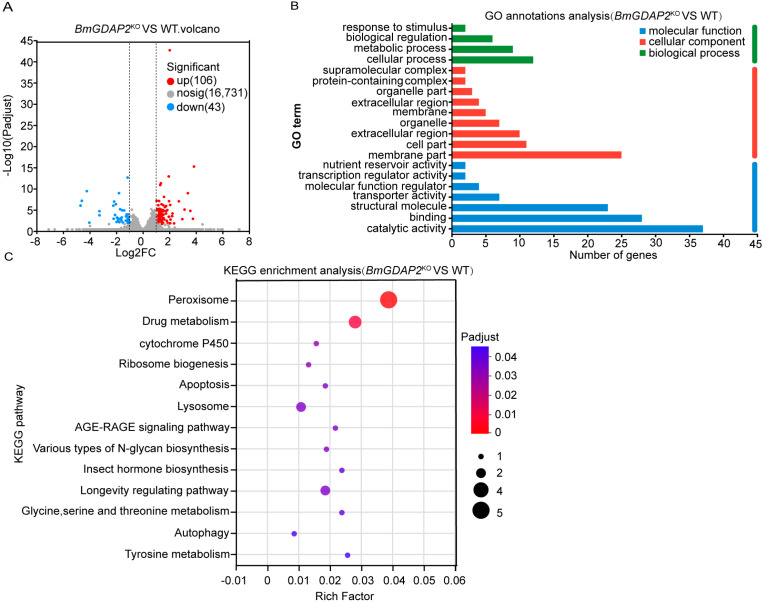
Differentially expressed genes and enriched pathways at the transcriptome level after knocking out *BmGDAP2*. (**A**) Statistical volcano plot of DEGs between *BmGDAP2*^KO^ and wild-type. Significant differential expression was determined using thresholds of Log2|Fold Change| ≥ 1 and Padjust < 0.05, with red indicating upregulated genes, blue representing downregulated genes, and gray denoting non-significantly modulated genes. (**B**) Bar chart illustrating GO annotation analysis of DEGs between *BmGDAP2*^KO^ and wild-type. The “Number of genes” represents the count of DEGs involved in each biological pathway. (**C**) Scatter plot of enriched KEGG pathways for DEGs. Rich factor denotes the ratio of DEGs to total annotated genes in a specific pathway. Significantly enriched KEGG pathways (Padjust < 0.05) are presented, with the point size reflecting the number of associated DEGs and the color intensity indicating the enrichment significance.

In order to further explore the regulation network and possible molecular mechanisms of *BmGDAP2* in the development of silkworms, we performed comprehensive OVA comparative transcriptome analyses between the wild-type and the *BmGDAP2*^KO^ mutant with RNA-seq data for each sample. In total, there were 149 genes identified as differentially expressed genes (DEGs) in the *BmGDAP2*^KO^ mutants compared to the wild-type silkworms, with significantly more upregulated genes (149) than downregulated (43) (Figure 4A and Table S2). Gene ontology (GO) annotation analysis indicated that the common genes were enriched in biological processes and molecular functions, such as structural molecule activity, catalytic activity, and binding activity. These genes were also enriched in nutrient metabolism and catalytic processes (Figure 4B). In fact, they are annotated as Mitotic Arrest-Deficient 2 Like 1 (*MAD2L1*, KWMTBOMO12817) and Aurora Kinase B (*Aurka*-b, KWMTBOMO05416). KEGG enrichment analysis indicated that these DEGs were significantly enriched in pathways related to cell proliferation (Figure 4C), such as apoptosis and autophagic pathways, the lifespan regulation pathway, and the hormone synthesis pathway, which is important in larva development [26,27,28,29].

### 3.5. Candidate DEGs Involved in Larva Development in BmGDAP2^KO^ and Wild-Type Silkworms

**Figure 5:**
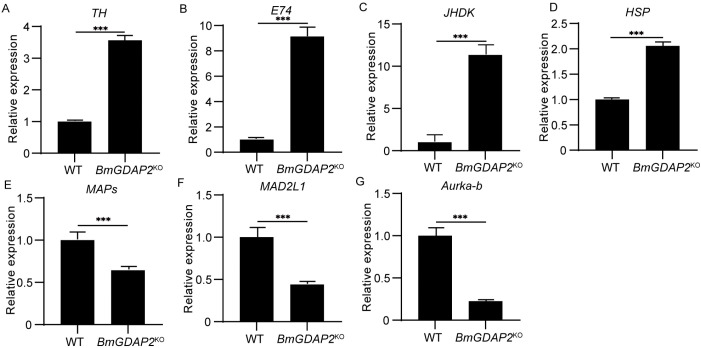
qRT-PCR validation of candidate differentially expressed genes involved in different pathways: (**A**) tyrosine metabolism pathways; (**B**,**C**) hormone regulatory pathways; (**D**) longevity regulatory pathways; (**E**) autophagy pathway; (**F**,**G**) cell division and proliferation. Values are represented as means ± S.E.s (error bars). For the significance test, *** *p* < 0.001.

To further elucidate DEGs significantly enriched in the functions of autophagy, apoptosis, and hormone biosynthesis between *BmGDAP2*^KO^ and wild-type silkworms, candidates were selected, including *TH* (tyrosine hydroxylase, KWMTBOMO00244), *E74* (transcription factor E74, KWMTBOMO08598), *JHDK* (juvenile hormone diol kinase, KWMTBOMO01580), *HSP* (19.5kDa heat shock protein, KWMTBOMO02376), *MAPs* (microtubule-associated protein 1 light chain 3, KWMTBOMO03983), *MAD2L1* (Mitotic Arrest-Deficient 2 Like 1, KWMTBOMO12817), and *Aurka*-b (Aurora Kinase B, KWMTBOMO05416) (Table S2). Because the phenotype of fifth-instar larvae is more pronounced, we chose this instar for qRT-PCR of DEGs. The results show that the expression levels of *TH* (Figure 5A), *E74* (Figure 5B), *JHDK* (Figure 5C), and *HSP* (Figure 5D) were significantly upregulated, while *MAPs* (Figure 5E), *MAD2L1* (Figure 5F), and *Aurka*-b (Figure 5G) were significantly downregulated. Previous studies have shown that *TH* is involved in insect melanin and catecholamine biosynthesis pathways; knocking out *TH* in *Agrotis ipsilon* leads to developmental arrest and even death [30], indicating that changes in *TH* expression can regulate the development of silkworm larvae. In *Drosophila*, pharmacological activation of the ecdysone receptor EcR significantly upregulates the expression of *E74*, improving the reproductive capacity and lifespan of aged flies [28]. *JHDK*, which degrades juvenile hormone (*JH*) in insects, is regulated in concert with juvenile hormone esterase (*JHE*) and juvenile hormone epoxide hydrolase (*JHEH*) [29]; the upregulation of *E74* and *JHDK*, which are related to hormone synthesis pathways, may affect silkworm development by influencing the levels of 20E and *JH*. These results suggest that the knockout of *BmGDAP2* may disrupt the balance of *JH*, leading to abnormal growth and development in *BmGDAP2*^KO^ larvae. *HSP* was first discovered in *Drosophila*, where it enhances the stress resistance of the organism, inhibiting normal cell death and thereby regulating the balance of cell survival and death. In previous studies, *HSP* knockout would cause cells to age rapidly, whereas *HSP* overexpression reduced senescence and prolonged lifespan [31]. Furthermore, we also found that genes involved in the lifespan regulation pathway, such as *HSP*, were significantly upregulated. Additionally, ATG8, a member of the autophagy-related microtubule-associated proteins in silkworms, is associated with the autophagy pathway. The downregulation of *MAPs* leads to a significant decrease in insect survival, resulting in abnormal or lethal phenotypes [26,27], indicating that changes in *MAPs* affect development by influencing autophagy. These pathway gene changes may also impact the expression of genes related to cell proliferation and division pathways. A decrease in the expression of *MAD2L1*, a crucial component of the mitotic checkpoint complex, suppresses cell proliferation and migration and promotes apoptosis [32,33]. *Aurka*-b, a cyclin-regulated serine/threonine kinase that plays an important role in mitosis, phosphorylates multiple protein substrates essential for mitosis. Therefore, inhibition of *Aurka*-b disrupts mitotic progression, thereby impairing cell proliferation [34]. Previous studies have reported that *GDAP2* is involved in cell replication, and we speculate that *BmGDAP2* knockout decreases the expression of *MAD2L1* and *Aurka*-b, which in turn affects cell replication and individual development.

### 3.6. BmGDAP2 Mainly Regulates Development Through the Peroxisome Pathway

**Figure 6:**
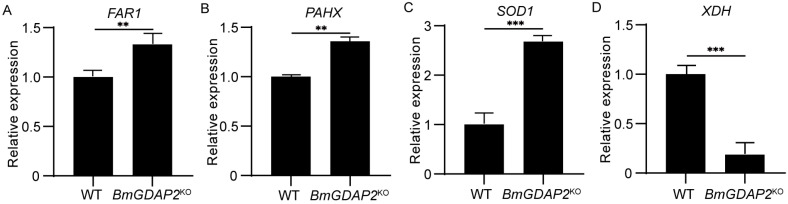
qRT-PCR validation of genes in the peroxidase pathway. (**A**) qRT-PCR of genes related to lipid metabolism. (**B**) qRT-PCR of genes related to phytanoyl-CoA metabolism. (**C**) qRT-PCR of genes related to the metabolism of superoxide anion radicals. (**D**) qRT-PCR of genes related to purine metabolism. Values are represented as means ± S.E.s (error bars). For the significance test, ** *p* < 0.01, and *** *p* < 0.001.

Previous studies have reported that genes involved in the peroxisome pathway, such as those affecting purine, superoxide anion radicals, and lipid metabolism, may disrupt metabolic homeostasis when dysregulated. At the same time, they may slow down aging [35,36,37,38], thereby affecting normal growth and development. The peroxisome pathway was significantly enriched in the DEGs; we speculated whether genes such as *FAR1* (fatty acyl-CoA reductase, KWMTBOMO14223), *PAHX* (phytanoyl-CoA dioxygenase, KWMTBOMO16177), *SOD1* (superoxide dismutase Cu-Zn 1, KWMTBOMO05949), and *XDH* (xanthine dehydrogenase, KWMTBOMO07216) (Table S2) were modulated by *BmGDAP2*. RT-qPCR analysis showed that the expression levels of *FAR1* (Figure 6A), *PAHX* (Figure 6B), and *SOD1* (Figure 6C) were significantly upregulated, while the expression of *XDH* (Figure 6D) was significantly downregulated. Fatty alcohols serve as critical components of insect sex pheromones. Fatty alcohols are components of sex pheromones in insects, and *FAR1* is an important enzyme needed to reduce fatty alcohols; changes in the expression level of this gene can lead to abnormal levels of fatty alcohols, which can lead to stunted development in insects [35,39]. Studied DEGs have shown that the knockout of *PAHX* leads to the abnormal development of silkworm eggs, so an increase in the expression level of this gene may lead to the abnormal development of silkworms [40]. At the same time, studies have also shown that increased *SOD1* expression levels can effectively remove ROS in the body, thereby delaying aging [37,38]. On the other hand, *XDH* catalyzes the oxidation of hypoxanthine and xanthine to uric acid during the catabolism of purine nucleotides and produces ROS in the process. Interestingly, when the expression of *XDH* is reduced, the production of ROS decreases, which may extend lifespan [36]. Based on these results, we propose that *BmGDAP2* might regulate silkworm growth and development through the coordinated modulation of peroxisomes.

## 4. Discussion

The life cycle of a silkworm is a complex and delicate developmental process regulated by a variety of internal and external factors, including hormones, gene expression, and environmental conditions [7,8,9,10]. In this study, we observed the phenotype of *Bombyx mori* with *BmGDAP2* knockout and found that *BmGDAP2*^KO^ had a longer growth cycle and smaller body size. It was preliminarily determined that *BmGDAP2* would inhibit the growth and development of silkworms. However, a shortcoming of this study is that *BmGDAP2*^KO^ was only compared with WT, not with the single-fluorescence type.

Transcriptomic profiling identified candidate genes, including *TH*; *TH* knockout has been shown to lead to developmental arrest and lethality, indicating its essential role in larval progression [30], suggesting that changes in *TH* expression can regulate the development of silkworm larvae. Additionally, Atg8, a microtubule-associated protein linked to autophagy, and *MAPs* showed significant downregulation, correlating with reduced survival rates [26,27]. *HSP* overexpression has been shown to reduce senescence and prolong lifespan [31]. Notably, *MAD2L1* and *Aurka*-b, involved in cell cycle regulation, exhibited altered expression levels. Reduced *MAD2L1* expression suppressed cell proliferation and promoted apoptosis [32,33], while inhibition of *Aurka*-b disrupts mitotic progression, thereby impairing cell proliferation [34]. Therefore, we speculate that *BmGDAP2* knockdown may affect the expression of *MAD2L1* and *Aurka*-b and then affect the cell division and proliferation of *Bombyx mori*. The transcription factor *E74*, which is related to hormone levels, is closely related to the role of 20E; the expression of *E74* was significantly upregulated by the drug activation of the ecdysone receptor EcR, which improved the reproductive ability and longevity of the elderly in *Drosophila* [28]. Concurrently, upregulated juvenile hormone diol kinase (*JHDK*), which degrades *JH*, caused *JH* level dysregulation, thereby prolonging larval development [29].

At the same time, we discovered the enrichment of genes related to the peroxidase pathway. Fatty alcohols are components of sex pheromones in insects, and *FAR1* is an important enzyme needed to reduce fatty alcohols; changes in the expression level of this gene can lead to abnormal levels of fatty alcohols, which can lead to stunted development in insects [35,39]. Studies have shown that *PAHX* knockout leads to the abnormal development of silkworm eggs [40]. To counteract the deleterious effects of ROS, *SOD1* serves as a critical regulator. The upregulation of *SOD1* expression significantly reduces ROS levels, thereby potentially influencing developmental processes and extending organismal lifespan [37,38]. On the other hand, *XDH* catalyzes the oxidation of hypoxanthine and xanthine to uric acid during purine nucleotide catabolism, generating ROS as a byproduct of this enzymatic cascade. Interestingly, the downregulation of *XDH* expression leads to diminished ROS production, suggesting a dual regulatory role of this enzyme in redox homeostasis [36].

Therefore, mutations in *BmGDAP2* may lead to slow development and an extended lifespan. This is mainly because this knockout affects autophagy and apoptosis, causing metabolic abnormalities; at the same time, hormone regulation becomes disrupted, cell division is inhibited, and there are abnormalities in genes related to the peroxisome pathway, all of which then affect the growth and development of the silkworm. In this study, we found that *BmGDAP2* may prolong the lifespan of silkworm larvae, but the specific mechanism needs further verification.

Previous studies indicate that the downregulation of *GDAP2*, a member of the *GDAP* family, is associated with cerebellar ataxia in mammals [14,15]. In silkworms, *GDAP2* has been implicated in neural signaling; however, the pronounced developmental delay in knockout strains precluded deeper neurofunctional analysis. Comparative studies revealed elevated *GDAP2* expression in the silk glands of high-silk-yielding domesticated silkworms versus their wild counterparts, suggesting a role in silk protein synthesis [18]. Phenotypic analysis of *GDAP2*-knockout silkworms demonstrated stunted silk gland development, supporting its potential regulatory role in silk gland morphogenesis. Nevertheless, the mechanistic basis remains unclear. Future research will further investigate the specific mechanisms by which this gene regulates growth and development.

In conclusion, mutations in *BmGDAP2* may significantly prolong the growth cycle of *BmGDAP2*^KO^ larvae while reducing their body size. Pathways and genes related to development were enriched through transcriptome sequencing. The discovery of the function of *GDAP2* in insect development not only provides a new target for the study of the insect life cycle but also provides new materials for the study of insect neural regulation.

## Data Availability

The original contributions presented in this study are included in the article/Supplementary Materials; further inquiries can be directed to the corresponding author/s. Transcriptome data: The raw data have been deposited in the National Center for Biotechnology Information (NCBI) database under the BioProject number PRJNA1214548.

## Supplementary Materials

The following supporting information can be downloaded at https://www.mdpi.com/article/10.3390/insects16040354/s1. Figure S1. N4 strains (pBac [IE1-EGFP-Nos-Cas9]) were selected by screening for green fluorescence markers in the segments of the moths. Figure S2. Hybridization of *BmGDAP2*_gRNA with Cas9. The following four situations were obtained: non-fluorescent, green-fluorescent eyes, green-fluorescent body, and dual fluorescence (*BmGDAP2*^KO^). Table S1. Primers used in this study. Table S2. RNA-seq of candidate DEGs. Supplementary Data S1. Detailed information for all DEGs. Supplementary Data S2. GO annotation analysis of DEGs. Supplementary Data S3. KEGG pathway analysis of DEGs.
