# Supplementary material for: CRISPR/Cas9-Mediated Knockout of BmGDAP2 in the Silkworm, Bombyx mori: Extended Lifespan and Altered Gene Expression Impacting Developmental Pathways
Source: Insects. 2025 Mar 27;16(4):354. doi: 10.3390/insects16040354 (PMC12028214; doi:10.3390/insects16040354)
Supplement: Supplementary file 1 [file insects-16-00354-s001.zip › insects-3487656-supplementary/Figure S2.pdf]

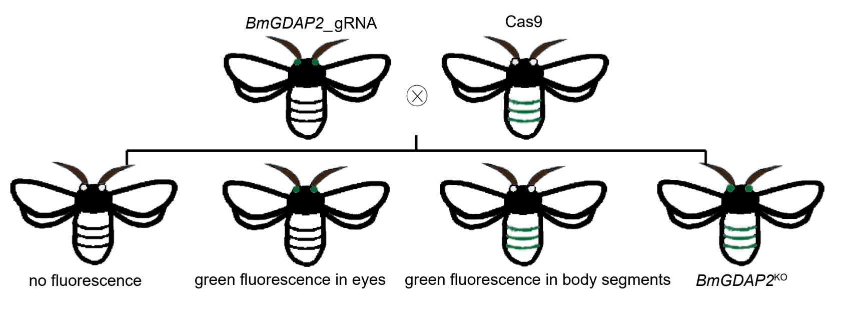

BmGDAP2_gRNA
Cas9
no fluorescence
green fluorescence in eyes
green fluorescence in body segments
BmGDAP2KO
