# Supplementary material for: CRISPR/Cas9-Mediated Knockout of BmGDAP2 in the Silkworm, Bombyx mori: Extended Lifespan and Altered Gene Expression Impacting Developmental Pathways
Source: Insects. 2025 Mar 27;16(4):354. doi: 10.3390/insects16040354 (PMC12028214; doi:10.3390/insects16040354)
Supplement: Supplementary file 1 [file insects-16-00354-s001.zip › insects-3487656-supplementary/Table S1.pdf]

Table S1. Primers used in this study.

Gene	Accession number	Primer sequences(5'-3')
BmGDAP2	XP_062525983	F:TCTGGACTAAGATGATGACGTG
		R:GAGATCACGGACATTTGTTCTG
XDH	NP_001037333	F:TAGTTGGTGCTGCCGTGACAT
		R:CGGCGAACCAGTGTAACATAGC
FAR1	XP_012545692	F:ACGGGCATCGAGAAACCACAA
		R:GATACCAGAGGCACACGGAGAA
PAHX	XP_062532757	F:ACATCTCCGCCGTCAATCTGAT
		R:CCGTCCACATCGCTATGATCTTG
SOD1	XP_062526713	F:TCCACCAAACGGGCGACATAA
		R:GAACGAGGTCCAGCCAACGA
TH	NP_001138794	F:AACTACAGGCAGCAGACATATTCG
		R:CAAGCGAGGCGAGGAAGTCA
MAPs	XP_00493134	F:GTGTTCGGCTACCAGGATGATATG
		R:TGGCACCTCACGATCTCTTACG
HSP	NP_001164470	F:GCTACGACACTTGAGGAATCTGG
		R:GTGGCTTGCCTGATGTACTTGAT
MAD2L1	XP_004925913	F:ATGAAATGGTCGGGAGCAAAGATT
		R:GGTTGTGCCTCAGCCCATTT
Aurka-b	NP_001274773	F:GCCAGTGACAACAAAGACCAGAC
		R:ACGGCGTACTTGGTGCTCTA
E74	XP_062528324	F:CCCGACGACATCCTGAAGCA
		R:GGCGACGATATGTTGTTGTTCTCT
JHDK	NP_001037080	F:AATGGACTCCAGAAGGCAGC
		R:CCGAGTCTTGGACGTGGAAA
sw22934		F:TTCGTACTGGCTCTTCTCGT
		R:CAAAGTTGATAGCAATTCCCT
BmGDAP2-sgRNA		F:AAGTGCCCTTTTCGTGGCTCAAGG
		R:AAACCCTTGAGCCACGAAAAGGGC
BmGDAP2^{KO}		F:ACCAGCCGCAGAAGAAACA
		R:CCTCCTTGAGCCACGAAAA
