# Supplementary material for: CRISPR/Cas9-Mediated Knockout of BmGDAP2 in the Silkworm, Bombyx mori: Extended Lifespan and Altered Gene Expression Impacting Developmental Pathways
Source: Insects. 2025 Mar 27;16(4):354. doi: 10.3390/insects16040354 (PMC12028214; doi:10.3390/insects16040354)
Supplement: Supplementary file 1 [file insects-16-00354-s001.zip › insects-3487656-supplementary/Table S2.pdf]

Table S2. RNA-seq of candidate DEGs

Gene_ID	Description	P value	$log_2(FC)$	Path	Regulation
KWMTBOMO07216	*XDH*	0.0003	-1.1955	Peroxisome	down
KWMTBOMO14223	*FAR1*	0.0004	1.2161	Peroxisome	up
KWMTBOMO16177	*PAHX*	4.26E-07	1.2372	Peroxisome	up
KWMTBOMO05949	*SOD1*	4.58E-09	1.5141	Peroxisome	up
KWMTBOMO00244	*TH*	2.30E-06	1.2045	Tyrosine metabolism	up
KWMTBOMO03983	*MAPs*	5.06E-05	-1.8791	Autophagy	down
KWMTBOMO02376	*HSP*	0.0001	1.5951	Longevity regulating pathway	up
KWMTBOMO12817	*MAD2L1*	1.11E-05	-1.2775		down
KWMTBOMO05416	*Aurka*-b	0.0011	-1.09542		down
KWMTBOMO08598	*E74*	0.0009	1.11820	Apoptosis	up
KWMTBOMO01580	*JHDK*	2.29E-10	1.02393	Insect hormone biosynthesis	up
