# Supplementary figures and images for: CRISPR/Cas9-Mediated Knockout of BmGDAP2 in the Silkworm, Bombyx mori: Extended Lifespan and Altered Gene Expression Impacting Developmental Pathways
Source: Insects. 2025 Mar 27;16(4):354. doi: 10.3390/insects16040354 (PMC12028214; doi:10.3390/insects16040354)

BF

EGFP

WT

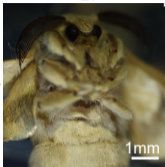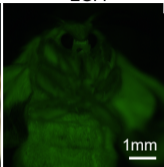

Cas9

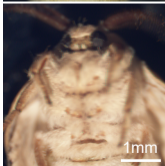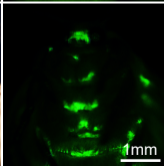

Supplement: Supplementary file 1 [file insects-16-00354-s001.zip › insects-3487656-supplementary/Figure S1.pdf]
